# Comparative Transcriptome Analysis of *Eriocheir sinensis* from Wild Habitats in Han River, Korea

**DOI:** 10.3390/life12122027

**Published:** 2022-12-05

**Authors:** Hyung-Eun An, Tae-June Choi, Chang-Bae Kim

**Affiliations:** Department of Biotechnology, Sangmyung University, Seoul 03016, Republic of Korea

**Keywords:** *Eriocheir sinensis*, transcriptome, RNA-Seq, differential expressed genes, wild habitat

## Abstract

*Eriocheir sinensis* is an euryhaline crab found from East Asia to Europe and North America. This species can live in freshwater and seawater due to the unique physiological characteristics of their life cycle, which allows them to adapt and inhabit different habitats in a wide range of environments. Despite the wealth of studies focusing on adaptation mechanism of *E. sinensis* to specific environmental factors, the adaptation mechanisms to wild habitats with coexisting environmental factors are not well understood. In this study, we conducted a transcriptome analysis to investigate gene expression differences related to habitat adaptation of *E. sinensis* from two wild habitats with different environmental factors in the Han River, Korea. A total of 138,261 unigenes were analyzed, of which 228 were analyzed as differentially expressed genes (DEGs) between the two wild habitats. Among 228 DEGs, 110 DEGs were annotated against databases; most DEGs were involved in energy metabolism, immunity, and osmoregulation. Moreover, DEG enrichment analysis showed that upregulated genes were related to biosynthesis, metabolism, and immunity in an habitat representing relatively high salinity whereas downregulated genes were related to ion transport and hypoxia response in habitats with relatively low salinity and dissolved oxygen. The present findings can serve as foundation for future *E. sinensis* culture or conservation approaches in natural conditions.

## 1. Introduction

*Eriocheir sinensis*, an euryhaline species, is one of the most important aquaculture crustaceans in Korea [1,2]. In this country, they are distributed along the West coast, including the Han and Geum rivers [3]. This species is also broadly distributed from the Eastern Pacific coast of China to the Korean Peninsula and found as an invasive species in Europe and the United States of America (USA) [4]. *E. sinensis* is unique among crustaceans as it requires two different environmental conditions during its life cycle [4]. Adults grow in freshwater, reproduction occurs in brackish water, and larvae hatch offshore [5]. In spring, the larvae gradually grow to larval crabs at the estuary, moving upstream and living in the freshwater environment until adulthood. As adults, they migrate to brine for reproduction [5]. This complex life cycle has made this species adapt and evolve to survive and reproduce in different habitats [6]. Habitats presenting a wide range of environmental factors can affect *E. sinensis* resulting in stress and the associated physiological responses during migration [7,8]. For instance, previous studies have reported that pH stress induced oxidative damage in *E. sinensis* [9]. Salinity is known to affect the growth and reproduction in *E. sinensis* [10].

RNA sequencing has been used to generate large amounts of transcript sequences and gene expression data for non-model species without sequenced genomes and has been widely applied to transcriptomics [11,12,13]. Since animal adaptability to habitats with different environmental factors largely depends on gene expression and transcriptome responses [14,15], numerous studies have investigated the adaptation mechanisms of *E. sinensis* to different environmental conditions by evaluating its transcriptome changes [5,16,17]. Among the tissues of *E. sinensis*, the gill is the first exposed to changes in the aquatic environment and the main respiration and osmotic regulation organ [18]. For example, the transcriptome from *E. sinensis* gills was analyzed to identify genes involved in osmoregulation at different salinity levels, and pH stress and immune-related genes were analyzed under air exposure stress in previous studies [19,20,21]. However, despite the numerous transcriptome studies performed on the gill of *E. sinensis* to understand its mechanisms of adaptation to specific environmental factors [4,5,20], the adaptation mechanisms to wild habitats with various coexisting environmental factors remain unknown. Studies have been conducted to understand the adaptation mechanisms of wild habitats from aquatic animals. The transcriptome analysis from Pacific sockeye salmon during spawning migration was analyzed to investigate environmental acclimatization and survival, and the genomic basis of adaptive response in variable osmoregulation from freshwater prawn was examined in wild habitats [22,23]. Therefore, to understand the adaptation mechanisms of *E. sinensis* in wild habitats, the transcriptome analysis in wild habitat is necessary.

In this study, transcriptome analysis was performed to investigate gene expression differences of *E. sinensis* living in two wild habitats with different environmental factors. *E. sinensis* were collected in two locations. Pocheon is upstream of the Han River water system and about 160 km away from the sea whereas Gimpo is downstream and close to the sea. We applied a transcriptomic approach (RNA sequencing) to compare the gene expression differences between *E. sinensis* found at the two locations, followed by differential gene expression (DEG), enrichment analysis of Gene Ontology (GO), Kyoto Gene and Genome Encyclopedia (KEGG) pathway as well as network analysis to infer the function of the detected DEGs. Network-based research provides a framework for obtaining a broad overview of data including multiple interacting groups. Therefore, we investigated and compared different sets of proteins and their interactions within the clusters identified in the network [24]. The results obtained in this study could suggest basic reference for aquaculture or conservation initiatives of *E. sinensis* in the wild.

## 2. Materials and Methods

### 2.1. Sample Collection

Nine healthy adult crabs were collected by local fishermen from Pocheon (PC) and Gimpo (GP) in Gyeonggi-do, Korea in October 2021 (Table S1), respectively. During sample collection, the environmental factors such as water temperature (WT), salinity, pH, and dissolved oxygen (DO) were measured using a portable meter (YK-2001PHA, LUTRON Co., Taipei City, Taiwan). The crabs were transported to the laboratory alive and allowed to stabilize for an hour. On the ice, fresh gill tissue was quickly collected from each crab and immediately stored into Qiagen RNAprotect tissue reagent (Qiagen, Valencia, CA, USA) at 4 °C overnight, and stored in −80 °C before further processing. The remaining specimens were stored in 99% ethanol.

### 2.2. Species Identification

Tissue from the pereopods in each specimen was used for species identification. DNA was extracted using a Qiagen DNeasy blood and tissue kit (Qiagen, Valencia, CA, USA) following manufacturer’s instructions. The concentration and purity of DNA samples were measured with a Maestro Nano spectrophotometer (Maestrogen, Hsinchu, Taiwan). A polymerase chain reaction was performed for the mitochondrial cytochrome c oxidase subunit I (*COI*) gene using an LCO1490/HCO2198 primer set [25] under the following conditions: an initial denaturation for 2 min at 95 °C, 35 cycles of 95 °C for 30 s, 48 °C for 45 s, and 72 °C for 1 min, and a final extension for 5 min at 72 °C. The amplified products were analyzed by electrophoresis in 1% (*w*/*v*) agarose gels in tris-acetate buffer. All animals were confirmed to be *E. sinensis* (accession numbers: OP143822~OP143839) based on morphological features and molecular phylogenetic analysis using the *COI* sequence. *E. sinensis* specimens were preserved in 99% absolute ethanol and deposited in the Department of Biotechnology, Sangmyung University, under voucher numbers SMU00200~SMU00217.

### 2.3. RNA Extraction

Samples were homogenized with mortar and pestle and total RNA was extracted from individual gill tissues using the Qiagen RNeasy Plus Universal Mini Kit (Qiagen, Valencia, CA, USA) following manufacturer’s instructions. RNA degradation and contamination were monitored on 1% agarose gels. RNA purity was evaluated using a NanoPhotometer spectrophotometer (Implen, Westlake Village, CA, USA). RNA concentration was measured using Qubit RNA Assay Kit in Qubit 2.0 Fluorometer (Life Technologies, Carlsbad, CA, USA). RNA integrity was assessed using the RNA Nano 6000 Assay Kit of the Agilent Bioanalyzer 2100 system (Agilent Technologies, Santa Clara, CA, USA). For each location, equal RNA amounts from three crabs were pooled together for creating the RNA-seq library and analyzed in triplicate.

### 2.4. cDNA Library and High Throughput Sequencing

RNA-seq library preparation and sequencing were carried out by Macrogen (Seoul, Republic of Korea). Briefly, libraries for each of the six samples were synthesized using the TruSeq Stranded mRNA Sample Preparation kit (Illumina, San Diego, CA, USA), according to manufacturer’s instructions. Paired-end sequencing (101 bp from each end) was then performed on the Novaseq (Illumina, San Diego, CA, USA) at a sequencing depth of 90–115 million reads per library. The raw reads quality was examined using FastQC v0.11.9 [26].

### 2.5. De Novo Transcriptome Assembly

Prior to assembly, the Trimmomatic [27] within the Trinity v.2.8.5 [28] was used to filter the reads under the following parameters: ILLUMINACLIP: TruSeq3-PE.fa:2:30:10 SLIDINGWINDOW:4:15 LEADING:5 TRAILING:5 MINLEN:25. Since the reference genome of *E. sinensis* [29] is incomplete, and annotation information was insufficient for further analysis, the RNA sequencing reads were assembled with a de novo approach. De novo transcriptome assembly on the resulting quality filtered reads was performed by Trinity v.2.8.5 using default parameters [28].

### 2.6. Assembly Assessment and Unigene Calculation

Assembly statistics were calculated using the TrinityStats.pl script provided within the Trinity pipeline. BUSCO v.5.3.2 [30] was used to assess the transcriptome completeness of all datasets against the Arthropoda dataset (Arthropoda Odb10), composed by 1013 single-copy ortholog genes with default parameters. Unigenes with 95% similarity were calculated using CD-Hit [31] to eliminate sequence redundancy. The reads were mapped back to the unigenes using RNA-Seq by Expectation Maximization (RSEM) [32].

### 2.7. Functional Annotation of Unigenes

Potential coding regions and open reading frames (ORFs) were predicted with the TransDecoder [33] pipeline using default parameters. Each ORF was ≥100 amino acids long and sequences < 100 residues were excluded. Unigenes were scanned against the NCBI GenBank UniProtKB/Swiss-Prot database [34] using BLASTp [35] with an e-value of 1e-5. The top hit for each query sequence was used for transcriptome annotation and subsequent characterization. BLASTp results were used to extract the associated Gene Ontology (GO) terms. Enrichment of GO terms, Pfam annotation, and Kyoto Encyclopedia of Genes and Genomes (KEGG) pathways of the unigenes were performed using ShinyGO v.0.61 [36] and PANTHER v17.0 [37] used for GO annotation, HMMER [38] used for Pfam annotation, and KEGG mapper [39] and KOBAS v3.0 [40] used for KEGG pathway annotation, respectively. Only enriched categories with a corrected *p*-value < 0.05 cutoff were selected. DEGs pathway and associated level of deregulation were visualized using the Pathview Web tool [41].

### 2.8. Differential Gene Expression Analysis

DEGs were identified based on read count data obtained from the analysis of gene expression levels. Gene expression levels were measured according to the Fragments Per Kilobase of transcript per million fragments mapped (FPKM) method. FPKM was used to standardize read count data and differences were analyzed using the DEseq2 [42] package for R v4.1.3. Filtering thresholds were *p*-value < 0.05 and fold change ≥ 2 (log2FC ≤ −1 or log2FC ≥ 1). To confirm the quality of the triplicated sample, Pearson correlation coefficient and principal component analysis (PCA) were performed with DEseq2 [42] and ggplot2 [43].

### 2.9. Network Analysis and Community Detection

The amino acid sequences of the DEGs were mapped to the STRING v.11.0 [44] database (STRING-DB) to predict interactions between DEGs using *Drosophila melanogaster* (fruit fly) as reference organism. The interaction network of the DEGs was visualized and further analyzed using Cytoscape v.3.8.0 [45]. Clusters with <5 nodes were excluded from the analysis. The node degree distribution and community structure were estimated using the Cytoscape’s NetworkAnalyzer [46] and GLay algorithm [47] plugins, respectively. The algorithm finds clusters by repetitiously discarding edges from the network and looking again for linked nodes [48]. Then, enrichment analysis for each cluster was performed to identify enriched GO terms and KEGG pathways associated with these clusters.

## 3. Results and Discussion

### 3.1. RNA Sequencing and De Novo Assembly

The Illumina Novaseq RNA sequencing platform generated 620,486,868 raw reads, of which 613,612,380 reads remained after trimming and low-quality filtering steps. A summary of RNA-seq data is presented in Table S2. Since the reference genome of *E. sinensis* [29] is incomplete, the RNA sequencing reads were assembled with a de novo approach. A total of 269,883 transcripts were generated after a de novo transcriptome assembly, whereby transcripts were calculated using CD-Hit based on 95% similarity for removing sequence redundancy [49]. Then, the longest transcript among the results of CD-Hit clustering was taken as unigene for subsequent analysis; 214,590 unigenes were obtained this way. The longest and shortest unigenes were 29,539 bp and 181 bp long, respectively. The mean GC content of unigenes was 45.1% (Table 1).

BUSCO analysis was performed to assess the completeness of the de novo assembly results (Table S3). Of the 1013 BUSCO groups searched, 95.85% of complete orthologs belonging to the phylum arthropods were detected from assembly results. As a result, the complete ratio was >95% indicating reliable assembly completeness [50].

### 3.2. Unigene Functional Annotation

For functional annotation, 214,590 unigenes were used as query sequences. TransDecoder identified 138,261 unigenes with the longest ORFs for unigene datasets. The annotation result and accession number of all unigenes are shown in Table S4. Among 138,261 unigenes, a BLASTp-based homology search for *E. sinensis* resulted in the annotation of 31,377 (22.69%) unigenes (Table S4), while 106,884 unigenes had no significant sequence homology against the NCBI UniProtKB/Swiss-Prot database. Among 138,261 unigenes, 1277 (0.92%) were assigned with GO annotation. We mapped 31,072 unigenes to the Pfam database using HMMER, and 1289 unigenes were mapped to KEGG annotations using KEGG mapper. A total of 35,014 unigenes were assigned to ≥1 database (Table 2).

### 3.3. DEGs and Functional Analysis

**Figure 1:**
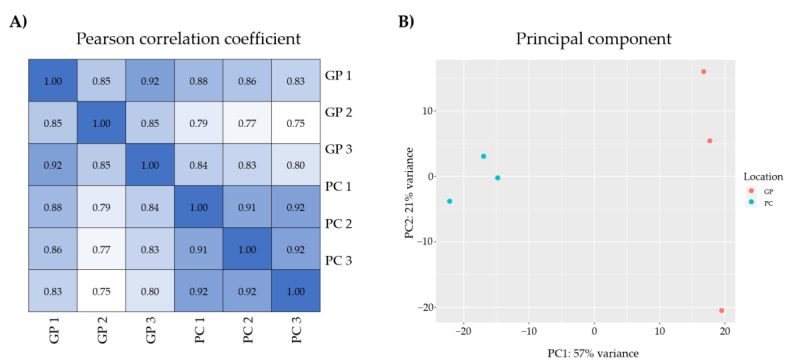
RNA-seq results: (**A**) Pearson correlation coefficient analysis between all biological replicated RNA-seq sample in *E. sinensis*; (**B**) principal component plot of biological RNA-seq samples in *E. sinensis*.

Pearson correlation coefficient and PCA was performed to confirm the deviation of triplicated samples by visualizing the differences in gene expression according to FPKM (Figure 1). Pearson correlation coefficient presented higher than 0.85 between samples from each location, and PCA result was clearly clustered according to locations (PC and GP). Accordingly, it identified that there was consistency between replicated samples. In this study, DEGs are genes whose expression was relatively compared between location PC and GP. Genes relatively highly expressed in samples collected from GP but not expressed in PC were defined as upregulated whereas genes highly expressed in samples collected from PC but not expressed in GP were considered downregulated. Compared with PC, a total of 228 DEGs, including 38 significantly upregulated genes and 190 downregulated genes were detected in GP (Table S5). Among 228 DEGs, only 110 DEGs (17 upregulated and 93 downregulated) were annotated and used for subsequent analyses.

**Figure 2:**
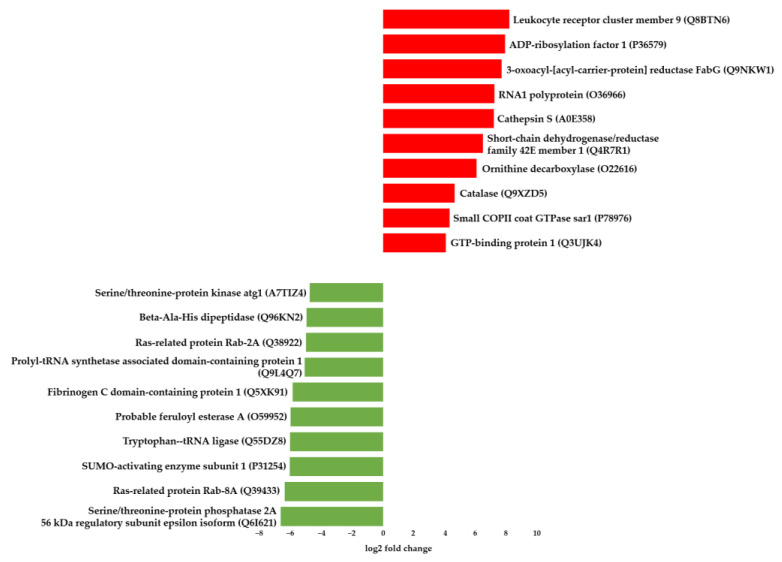
Top 10 upregulated and downregulated differentially expressed genes (DEGs) based on GP. Red and green bars indicate upregulated and downregulated genes, respectively. The graph was prepared according to the log2 fold change value; the gene name and Swiss-Prot accession number of the protein encoded by each gene are shown as well.

Figure 2 provides an overview of the top 10 upregulated and downregulated genes. The complete list with detailed annotations is provided in Table S5. The top 10 upregulated genes included two genes (Leukocyte receptor cluster member 9 (LENG9) and Catalase) related to metal binding, four genes related to ATP and GTP binding (ADP-ribosylation factor 1, RNA1 polyprotein, Small COPII coat GTPase sar1, and GTP-binding protein 1), one gene (3-oxoacyl-[acyl-carrier-protein] reductase FabG) related to the peroxisome, one gene (Cathepsin S) related to a thiol protease, one gene (short-chain dehydrogenase/reductase family 42E member 1) related to the membrane, and another (Ornithine decarboxylase) related to pyridoxal phosphate (Figure 2). From these data, LENG9 was the topmost upregulated gene. Its sequence in *E. sinensis* was previously described in a study on *E. sinensis* immunity [51]. However, the precise function of LENG9 in crabs remains unknown. In mice, LENG9 is highly abundant in macrophage associated organs, exhibiting similar expression patterns to other macrophage activation markers during macrophage activation [52]. These findings suggest a role for LENG9 in macrophage activation.

On the other hand, the top 10 downregulated genes comprised one gene related to salinity stress response (Serine/threonine-protein phosphatase 2A 56 kDa regulatory subunit epsilon isoform), five genes related to ATP and GTP binding (Ras-related protein Rab-8A, SUMO-activating enzyme subunit 1, Tryptophan-tRNA ligase, Ras-related protein Rab-2A, and Serine/threonine-protein kinase atg1), one gene (Probable feruloyl esterase A) that plays important roles in lipid metabolism, one gene (Prolyl-tRNA synthetase associated domain-containing protein 1) related to the cytoplasm, and two genes (Fibrinogen C domain-containing protein 1 and Beta-Ala-His dipeptidase) related to metal binding (Figure 1). The topmost downregulated gene encodes the Serine/threonine-protein phosphatase 2A 56 kDa regulatory subunit epsilon isoform. This protein is the B regulatory subunit of phosphatase 2A (PP2A), involved in salt stress response. Under salt stress conditions, it acts as a negative regulator of salt tolerance [34,53]. In this study, GP had relatively higher salinity than PC, suggesting that the expression of this gene was related to salinity.

Most top 10 upregulated and downregulated genes were associated with ATP and GTP binding, both related to energy metabolism [54]. Aside from the large amount of energy required for homeostasis regulation, crabs tend to expend even more energy under stress [21]. In the present study, most DEGs, such as Ras-related protein, SUMO-activating enzyme subunit 1, Tryptophan-tRNA ligase, Serine/threonine-protein kinase atg1, GTP-binding protein 1, Small COPII coat GTPase sar1, RNA1 polyprotein, and ADP-ribosylation factor 1 were related to energy metabolism, oxidative phosphorylation, and the Tricarboxylic acid (TCA) cycle pathways. Previous studies have shown that in *E. sinensis* osmoregulation depends on many genes including several ion transporters and ion transport channels involved in energy metabolism [4,5]. Moreover, the expression of metabolic genes such as those implied in the TCA cycle and glycolysis/gluconeogenesis in *E. sinensis* increased under pH stress [21]. The differences in environmental factors at the two habitats in this study might have activated the energy metabolism for adaptation through differential expression of genes related to ATP and GTP binding.

### 3.4. GO and KEGG Enrichment Analysis of DEGs

**Figure 3:**
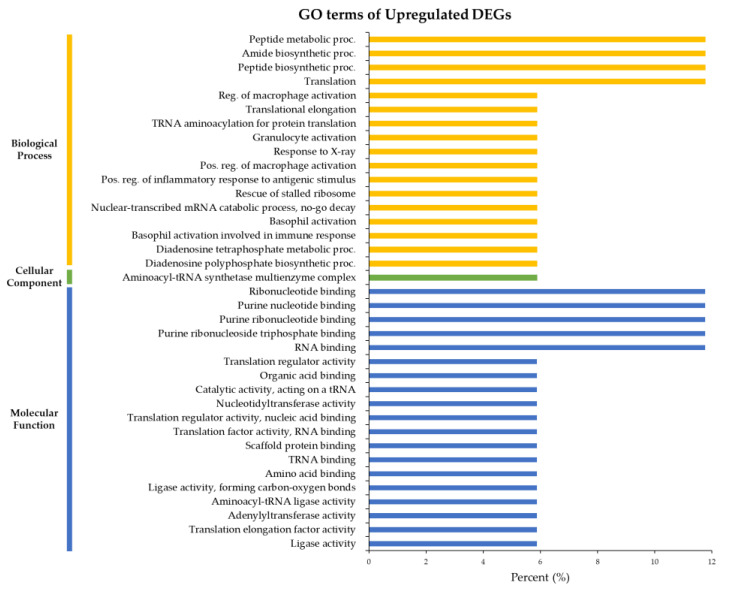
Gene ontology (GO) enrichment of upregulated genes based on GP. Yellow bars represent biological processes, green bars represent cellular components, and blue bars represent molecular functions, indicating the ratio of assigned genes to each GO term.

Enriched GO categories in terms of the biological process (BP), molecular function (MF), and cellular component (CC) associated with annotated DEGs were identified using the ShinyGO tool. To understand the difference in DEGs between PC and GP, the enriched GO terms were analyzed by dividing them into upregulated and downregulated genes. The 37 enriched GO terms of upregulated DEGs consisted of 17 BP, 1 CC, and 19 MF. “Peptide metabolic process”, “Amide biosynthetic process”, “Peptide biosynthetic process”, and “Translation” were the most common BPs (Figure 3). The GO term “Aminoacyl-tRNA synthetase multienzyme complex” was the most enriched CC (Figure 3) and “Ribonucleotide binding”, “Purine nucleotide binding”, “Purine ribonucleotide binding”, “Purine ribonucleoside triphosphate binding”, and “RNA binding” were the most enriched MFs (Figure 3).

**Figure 4:**
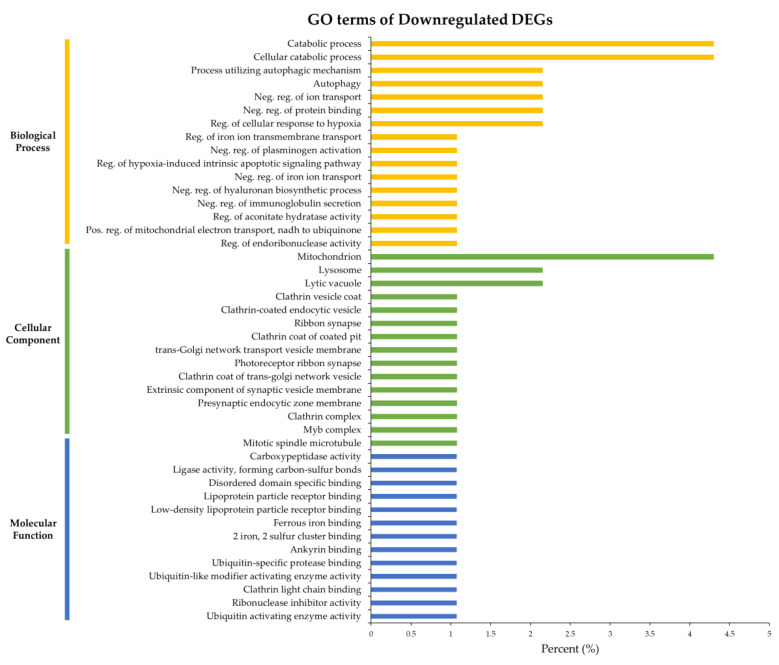
GO enrichment of downregulated genes based on GP. Yellow bars represent biological processes, green bars represent cellular components, and blue bars represent molecular functions, indicating the ratio of assigned genes to each GO term.

The 44 enriched GO terms of downregulated DEGs consisted of 16 BP, 15 CC, and 13 MF. “Catabolic process” and “Cellular catabolic process” were the most enriched BPs, in addition to “Process utilizing autophagic mechanism”, “Autophagy”, “Negative regulation of ion transport”, “Negative regulation of protein binding”, and “Regulation of cellular response to hypoxia” were enriched BP (Figure 4). The GO term “Mitochondrion” was the most enriched CC, but “Lysosome”, and “Lytic vacuole” were enriched as well (Figure 4). MFs were enriched for the same 13 GO terms (Figure 4), which were mostly related to binding.

GO analysis of enriched upregulated genes showed GO terms related to biosynthesis and metabolism. To understand the biological functions of those genes, we analyzed their function by focusing on GO terms corresponding to BPs. Upregulated genes were expressed in samples collected in relatively salinity environments. A previous study has reported that GO terms related to biosynthesis and metabolism are dominant in crabs exposed to high salinity [4]. Given the similarity of our findings, the activation of biosynthesis and metabolism might be closely related to salinity. In addition, upregulated genes showed GO terms related to immunity. According to previous studies, an increase in the number of blood cells may minimize and protect somatic cells against salt stress [55]. Some immune-related genes such as Heat shock protein 90 are related to pH and salinity stress [21]. Accordingly, the enriched immune-related GO terms found in this study may have been influenced by environmental factors such as high salinity and pH. On the other hand, the enriched GO terms of BPs of downregulated genes were related to ion transport and hypoxia, probably because PC samples were collected under relatively low salinity and DO conditions. According to previous research, low salinity induces ion loss through the body surface of *E. sinensis*, regulating it through cells specialized in ion exchange distributed in gill tissues [56]. It can induce apoptosis to protect cells from hypoxia and respond to hypoxic stress [19]. According to the present results, the low salinity and hypoxic state of the habitat influenced the transcriptome.

Using the KOBAS tool, the results of KEGG enrichment analysis associated with annotated DEGs showed 12 statistically significant pathways (1 up-regulated and 11 down-regulated) to be the most important in the DEGs. Most of these abundant pathways are related to metabolism and biosynthesis, similar to GO results (Table 3). Five DEGs were mapped to these pathways, one upregulated and the remaining four downregulated. The most enriched pathway was related to protein processing in the endoplasmic reticulum (Table 3). Two DEGs were mapped to this pathway (Figure S1): an upregulated (Heat shock protein HSP 90-alpha) and a downregulated gene (Alpha-crystallin B chain) were analyzed. This pathway is composed of genes involved in protein processing, and the genes also act to respond to stress and protect body cells (Table 3) [39]. The upregulated heat shock proteins involved in protein processing in endoplasmic reticulum metabolic pathway function as molecular chaperones that promote maturation, conformational maintenance, and proper regulation of specific target proteins involved in cell cycle control and signal transduction [34]. Similarly, the downregulated alpha-crystallin B chain acts as a chaperone to prevent the aggregation of various proteins under a wide range of stress conditions [34]. A meta-analysis using gill transcripts of *E. sinensis* also reported similar results, in which protein processing in the endoplasmic reticulum appeared as a statistically significant KEGG pathway [4]. In this study, the metabolic pathway including proteins named Protein henna and Homogentisate 1,2-dioxygenase (both related to iron, metal binding, and phenylalanine catabolism) and the spliceosome pathway, including the protein named Eukaryotic initiation factor 4A-III (involved in ATP binding and mRNA splicing) were statistically significant. Similarly, a previous meta-analysis using gill transcripts of *E. sinensis* determined that the metabolic pathways and the spliceosome were significant KEGG pathways [4]. Eukaryotic initiation factor 4A-III (EIF4A3) was related to various pathways including mRNA surveillance pathway (Figure S2) and nucleocytoplasmic transport (Figure S3). EIF4A3 was implicated in a number of cellular processes involving alteration of RNA secondary structure, such as translation initiation, nuclear and mitochondrial splicing, and ribosome and spliceosome assembly [57]. A previous study reported that this gene was downregulated in mussels in response to salinity stress [58]. In *E. sinensis*, further studies need to be performed for the EIF4A3 gene, but there is the probability that the EIF4A3 gene could be regulated by salinity stress.

### 3.5. Interaction Network of DEGs

**Figure 5:**
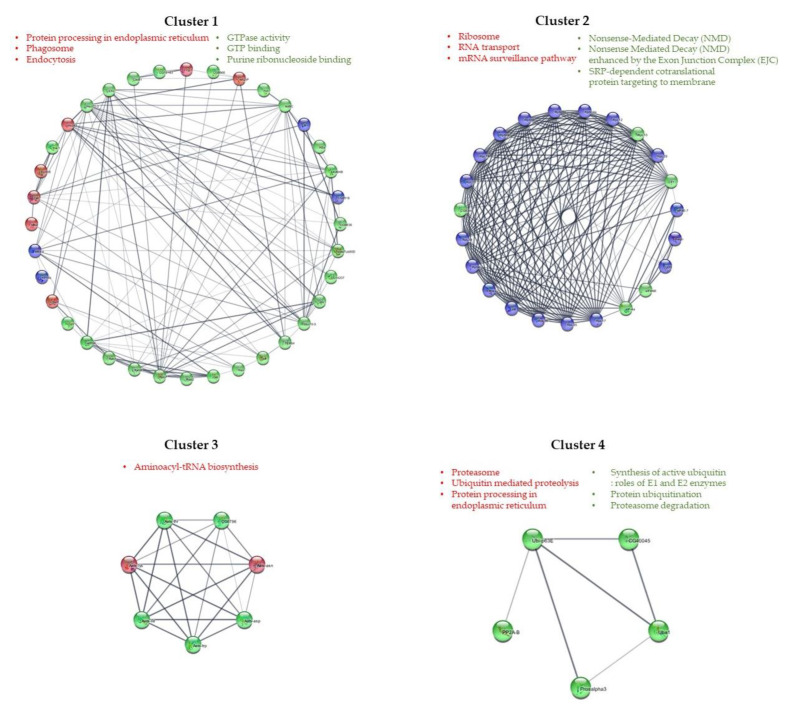
Community analysis of the interaction network. Red, green and blue nodes represent upregulated, downregulated genes and STRING-predicted genes, respectively. Abbreviations of the genes are retrieved from STRING-DB using *Drosophila melanogaster* as reference organism. The topmost enriched KEGG pathways (red text), GO terms (green text) are mentioned for each cluster. Clusters with <5 nodes were excluded from the analysis.

Network analysis was performed to predict the interactions between DEGs using 228 amino acid sequences. Of 228 DEGs, only 51 amino acid sequences (10 upregulated and 41 downregulated) were mapped to the fruit fly *D. melanogaster* proteome, whereas an additional 34 proteins were predicted as their interacting partners. The interaction network consisted of 85 nodes, each representing a gene, and 334 edges (node interactions; Figure 5). The node degree of each node in the network ranged between 1 and 20 including predicted genes. Among DEGs, the topmost two nodes representing the 16th and 19th node degree values were regarded as hubs from each cluster. These two nodes were downregulated transcripts in GP (downstream) and encoded the Heat shock protein 70 cognate 3 (node degree = 16) and Elongation factor 1-alpha 1 (node degree = 19). With respect to the first, Heat shock protein 70 cognate 3 enables several functions, including ATP binding activity whereas Elongation factor 1-alpha 1 promotes the GTP-dependent binding of aminoacyl-tRNA to the A-site of ribosomes during protein biosynthesis. Previous studies have shown that salt stress, affects the expression of heat shock protein 70 in crabs including *E. sinensis* [59,60]. Expression of heat shock protein 70 is presumed to be regulated to adapt to salinity by *E. sinensis*.

The interaction network was divided into four clusters (Figure 5). The enrichment analysis of all clusters suggested that each had specific functional roles. Among the largest clusters, in cluster 1, the enriched GO processes were related to GTPase activity while in cluster 2, they were related to nonsense-mediated decay. In contrast, the smallest cluster, cluster 4 showed functions related to the synthesis of active ubiquitin, among others. In addition, the enriched KEGG pathways in cluster 1 were related to the protein processing in endoplasmic reticulum while in cluster 2 was related to the ribosome, among others. Cluster 3 was related to aminoacyl-tRNA-biosynthesis. These results are consistent with previously analyzed GO and KEGG pathway analysis results; *E. sinensis* appears to adapt to different environments by regulating genes related to energy metabolism, immunity, and osmoregulation.

## 4. Conclusions

This study represented a preliminary analysis of the molecular mechanisms underlying the adaptation of *E. sinensis* to two wild habitats presenting a wide range of environmental factors in Korea. We obtained over 600 million clean reads and could identify DEGs related to metabolism, immunity, and osmoregulation between the two habitats. In the future, to better understand the relationship between wild habitats including environmental factors and difference of gene expression and identify the core genes related to environmental factors, it is necessary to add more locations and measure a wider range of environmental factors. Moreover, to clarify the environmental condition of the collection sites, environmental factors including substrate condition should be monitored more than once in each sampling site. In addition, in future studies, quantitative PCR should be performed on differentially expressed genes to find candidate genes and quantify the expression that clarify the differences between each habitat. The present results suggest a relationship between wild habitats and gene expression differences in *E. sinensis* which can inform *E. sinensis* culture or conservation strategies in real environments. Furthermore, our findings can facilitate further research into the functional genomics of this and other closely related species such as *E. japonica*.

## Figures and Tables

**Table 1:** Transcriptome datasets statistics and assembly.

Description	Statistics
Number of transcripts	269,883
Percent GC content (%)	45.4
Contig N50	1732
Median contig length (bp)	476
Average contig length (bp)	952.39
Total assembled bases (bp)	257,034,330
Total number of unigenes	214,590
Percent unigenes GC content (%)	45.1
Longest unigene (bp)	29,539
Shortest unigene (bp)	181

N50, Transcripts, for which the sum of transcript’s length was not less than 50% of the total length of mosaic transcripts sorted by length.

**Table 2:** Summary of unigene annotation results.

Description	Number of Unigenes	Percentage (%)
Total number of longest ORF unigene	138,261	
Annotated in Swiss-Prot	31,377	22.69
Annotated in GO	1277	0.92
Annotated in Pfam	31,072	22.47
Annotated in KEGG	1289	0.93
Total of annotated unigene	35,014	

**Table 3:** Enriched Kyoto Encyclopedia of Genes and Genomes pathways detected in DEGs identified by RNA-Seq of *E. sinensis* gills.

Regulation	Pathway Name	Pathway ID	Corrected *p*-Value
Up	Protein processing in endoplasmic reticulum	dme04141	0.01037
Down	Phenylalanine, tyrosine, and tryptophan biosynthesis	dme00400	0.01577
	Phenylalanine metabolism	dme00360	0.01577
	Tyrosine metabolism	dme00350	0.02671
	Folate biosynthesis	dme00790	0.03214
	Biosynthesis of amino acids	dme01230	0.03738
	Longevity regulating pathway—multiple species	dme04213	0.03738
	mRNA surveillance pathway	dme03015	0.03738
	Spliceosome	dme03040	0.04312
	Protein processing in endoplasmic reticulum	dme04141	0.04312
	RNA transport	dme03013	0.04312
	Metabolic pathways	dme01100	0.04312

## Data Availability

Sequence data generated and/or analyzed from this study are available on the Sequence Read Archive (SRA) SRR21780636~SRR21780641 under BioProject PRJNA886396.

## Supplementary Materials

The following supporting information can be downloaded at: https://www.mdpi.com/article/10.3390/life12122027/s1. Table S1: Environmental factors at each collection site (WT, water temperature; DO, dissolved oxygen). Table S2: Statistics for dataset from the *E. sinensis* gill transcriptome. Table S3: Results of BUSCO analysis. Table S4: Annotation results of unigenes. Table S5: Annotation results of DEGs. Figure S1: Pathway analysis of the protein processing in the endoplasmic reticulum using the Pathview. Highlighted genes that up or downregulated are components of these pathways significantly. Figure S2: Pathway analysis of the mRNA surveillance pathway using the Pathview. Highlighted genes that up or downregulated are components of these pathways significantly. Figure S3: Pathway analysis of the nucleocytoplasmic transport using the Pathview. Highlighted genes that up or downregulated are components of these pathways significantly.
