# Supplementary figures and images for: Comparative Transcriptome Analysis of Eriocheir sinensis from Wild Habitats in Han River, Korea
Source: Life (Basel). 2022 Dec 5;12(12):2027. doi: 10.3390/life12122027 (PMC9781331; doi:10.3390/life12122027)

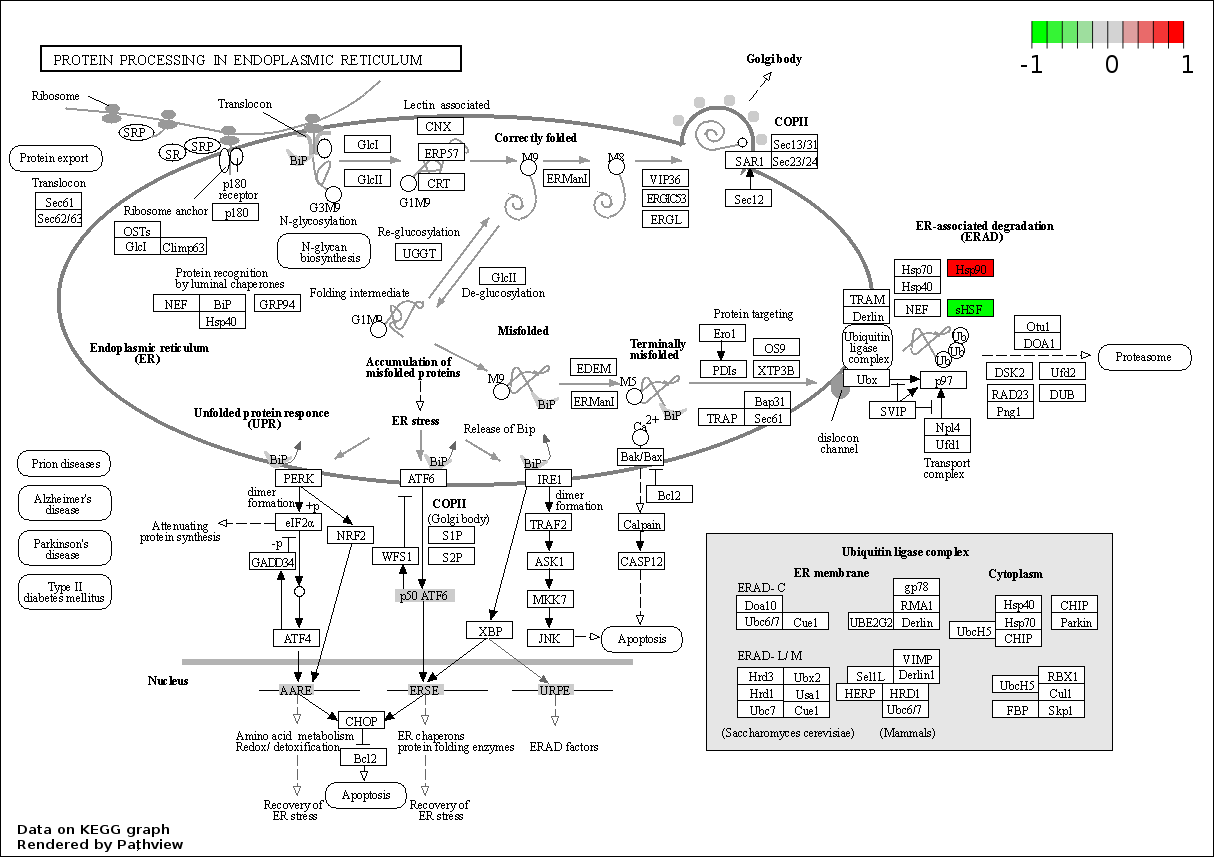

Supplement: Supplementary file 1 [file life-12-02027-s001.zip › Supplementary Figure S1.png]

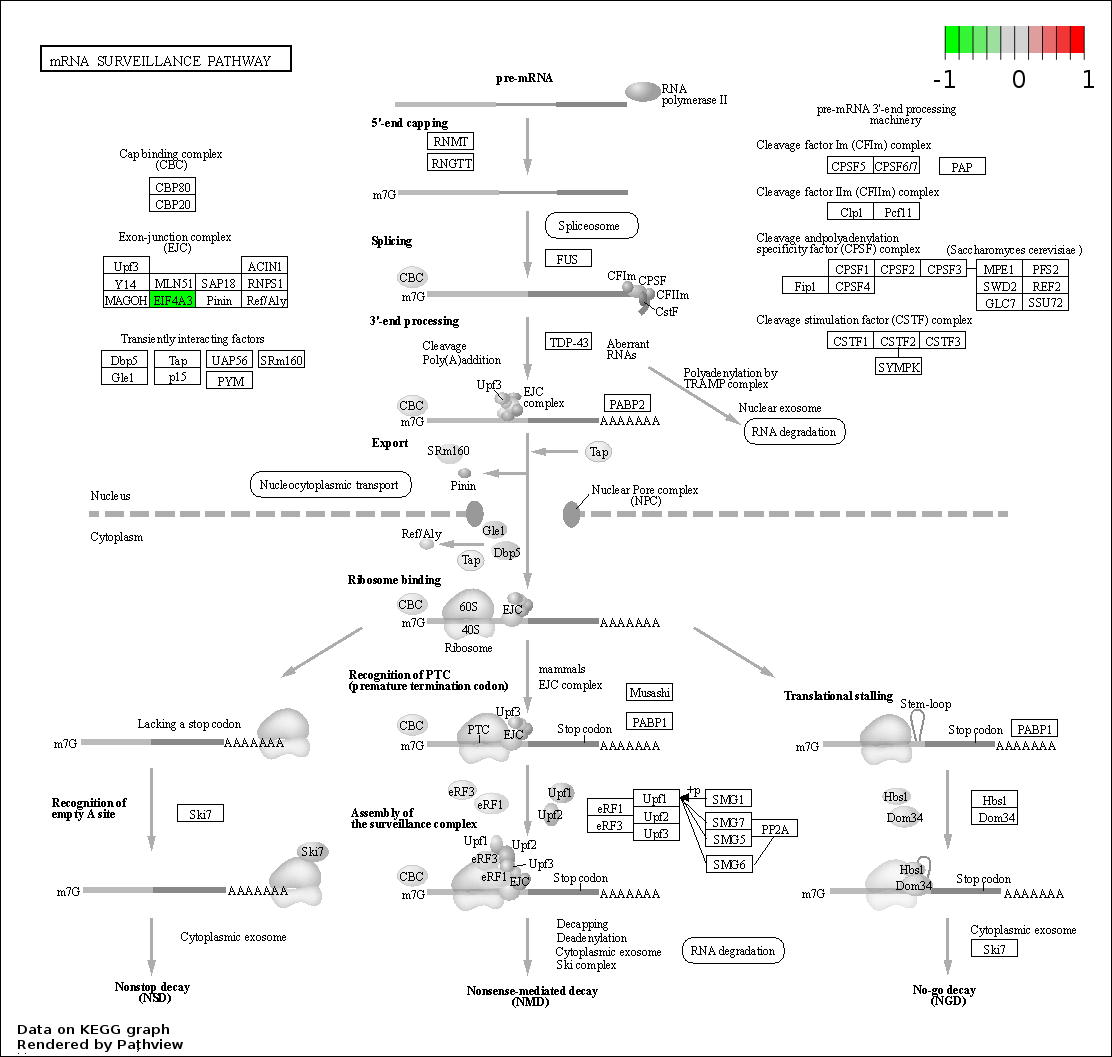

Supplement: Supplementary file 1 [file life-12-02027-s001.zip › Supplementary Figure S2.png]

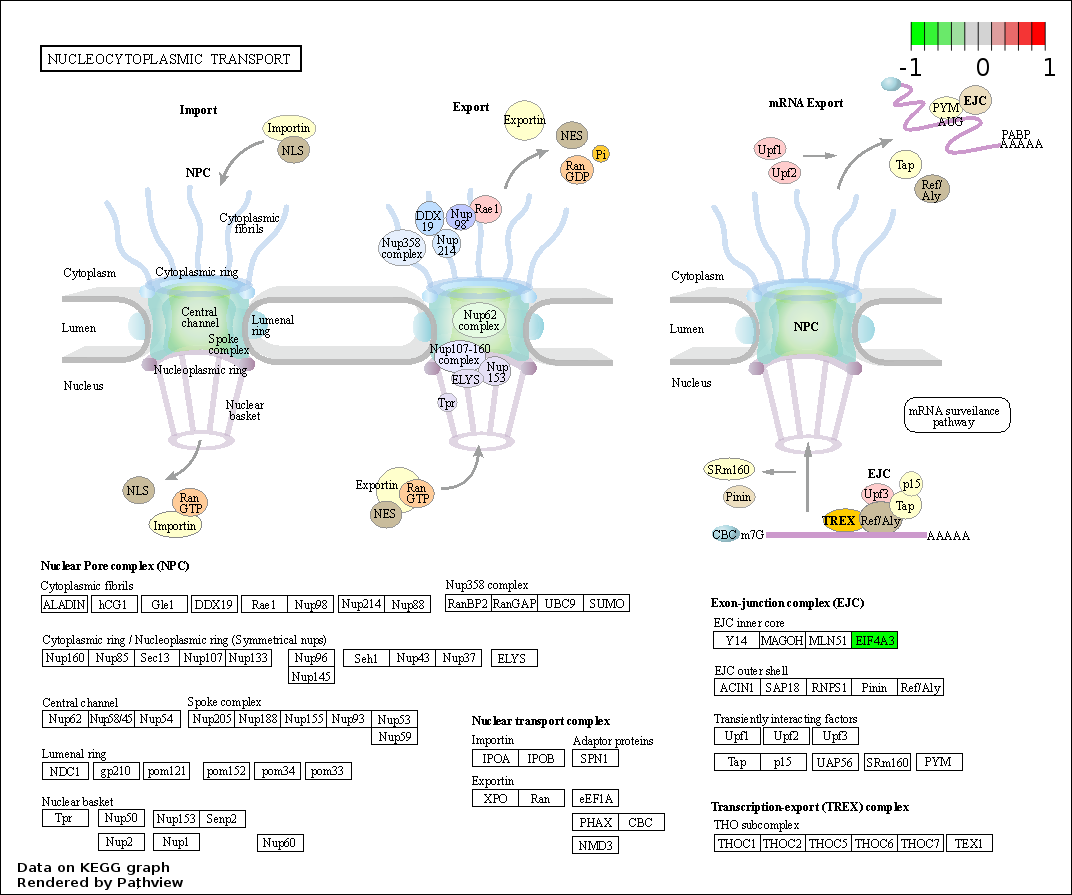

Supplement: Supplementary file 1 [file life-12-02027-s001.zip › Supplementary Figure S3.png]
